# Physiological Reports: A remarkable 10‐year journey

**DOI:** 10.14814/phy2.15888

**Published:** 2023-12-20

**Authors:** Thomas R. Kleyman

**Affiliations:** ^1^ Departments of Medicine, Cell Biology, and Pharmacology and Chemical Biology University of Pittsburgh Pittsburgh Pennsylvania USA

In the early 2010s the American Physiological Society and the Physiological Society began independent conversations about starting an open access journal. Fortunately, the societies decided to work together to jointly publish a new journal, leading to the founding of Physiological Reports in 2013 with Wiley as the journal's publisher. Sue Wray was selected as the journal's first Editor‐in‐Chief and I was fortunate to be asked to join as Deputy Editor‐in‐Chief, along with a talented group of associate editors. Key publication staff at the American Physiological Society, the Physiological Society, and Wiley had critical roles in launching the journal and in working with the editors in managing the journal. Following Sue Wray's 5‐year term as Editor‐in‐Chief, I transitioned to Editor‐in‐Chief for 5 years, working closely with Morten Thomsen as Deputy Editor‐in‐Chief and a new group of gifted associate editors.

A key founding tenant of Physiological Reports, which has continued to the present time, is that the journal would publish work that is scientifically sound. Manuscripts did not have to focus on original findings and we encouraged submissions of manuscripts that were confirmatory in nature. Furthermore, work could be primarily descriptive and did not have to address mechanisms. We believed that the journal should provide an outlet for investigators to publish scientifically sound work that may have difficulty in finding a home in other physiological journals. Based on our editorial philosophy we did not anticipate that the journal would have a high impact factor and we were pleasantly surprised when the journal received its first impact factor of 2.5 this past year. The journal's editorial leadership has strived to limit increases in publication fees, with the goal of preventing financial barriers to publishing in Physiological Reports.

Since its founding, authors were encouraged to directly submit their work to the journal. In addition, a cascade system was established whereby manuscripts rejected by a journal published by the American Physiological Society or the Physiological Society could be referred to Physiological Reports and, at the discretion of the authors, transferred to Physiological Reports along with the critiques, allowing our editors to handle these manuscripts in an expedited fashion. We have expanded the number of partner journals participating the cascade referral of manuscripts to 19. Over the past decade (prior to COVID), Sue, Morten, or I regularly met with the editorial teams of key partner journals to discuss the cascade process. We have a relatively user‐friendly system for transferring manuscripts from our partner journals to Physiological Reports and we appreciate the support that we have received from these journals in growing the number of manuscripts transferred to Physiological Reports.

As editors, we are proud of the journal's remarkable success over its first 10 years. Submissions have generally increased year to year, with a growing number of de novo submissions. We hope to see this trend continue under the editorial leadership of Jo Adams and her team. Over the past several years Physiological Reports has published brief, focused review articles and we anticipate the number of reviews published by the journal will continue to increase. During Sue's tenure as editor, we began to publish clinical case reports that have a physiologic focus. The journal has partnered with other society journals in issuing calls for papers on specific topics, including a very successful joint call for papers focusing on COVID‐19.

There are many challenges editorial teams face in starting a new journal. Perhaps the most challenging for Physiological Reports was for the physiological community to have a level of comfort in sending their work to the journal. Marketing efforts and interfacing with interest groups and sections within the American Physiological Society and with scientists at Physiological Society and Experimental Biology meetings was very important in the early years in encouraging authors to submit their work to the journal. It is been gratifying to see Physiological Reports transition from a startup to its role as an established journal within the physiological community. We look forward to the journal's continuing evolution and growth under Jo Adams leadership.

